# The impact of psychiatry school on attitudes towards psychiatry in medical students and junior doctors in Pakistan

**DOI:** 10.1192/bjo.2021.71

**Published:** 2021-06-18

**Authors:** Raja Adnan Ahmed, Sanaa Moledina, Usama Asad

**Affiliations:** 1 Cwm Taf Morgannwg University Health Board; 2 Northamptonshire Healthcare NHS Foundation Trust; 3 Shaheed Mohtarma Benazir Bhutto Institute of Trauma

## Abstract

**Aims:**

To assess the impact of Psychiatry School on the attitudes towards psychiatry in Pakistani medical students (MS) and junior doctors (JD).

**Method:**

Inspired by the Royal College of Psychiatrists’ ‘Choose Psychiatry’ campaign, an online event by the name of ‘Psychiatry Autumn School Pakistan’ was held on the 1st of November 2020. The event was promoted through social media and medical students and junior doctors from across Pakistan were invited to attend. Moreover, a panel of British and Pakistani psychiatrists belonging to different sub-specialties was invited to deliver talks. The attendees were provided an insight into psychiatry as a viable career option and were introduced to the training pathways, research opportunities, and the various sub-specialties present within the field.

Participants were requested to complete the 'Attitudes Towards Psychiatry' (ATP-30) questionnaires before and immediately after the event. Individual scores on the questionnaire can range from 30 to 150 and a high score indicates a positive attitude. Statistical analysis was performed using a paired t-test.

**Result:**

41 attendees (MS = 30, JD = 11) completed the pre-and post-school survey. The respondents were majorly female (76%) and from public sector universities (76%), with an average age of 23 years. The mean ATP score before the course was 119 (MS = 117, JD = 121) which increased by 9 points to 128 (MS = 126, JD = 131) after the event. When the two samples were compared using a paired t-test, the difference was statistically significant p < 0.005.

**Conclusion:**

We conclude that a psychiatry school can positively influence attitudes towards psychiatry in medical students and junior doctors and our findings are consistent with similar studies done in other countries.

In Pakistan, unfortunately, only 2–4% of undergraduate students opt for a career in psychiatry owing to insufficient knowledge and awareness about the available treatment modalities and advancement in the field. Hence, such an intervention can greatly enhance recruitment within the profession as it makes psychiatry more accessible and visible as a career choice, generates awareness about the effectiveness and evolution of psychotherapeutic practices, and eradicates misconceptions about the field that prevail among young doctors.

This was the first psychiatry school held in Pakistan and the findings of the study as well the feedback received from the participants and the speakers motivate us to continue campaigning for ‘Choose Psychiatry.’

